# Isolation and Identification of Uranium Tolerant Phosphate-Solubilizing *Bacillus* spp. and Their Synergistic Strategies to U(VI) Immobilization

**DOI:** 10.3389/fmicb.2021.676391

**Published:** 2021-07-13

**Authors:** Juan Zhong, Xuewu Hu, Xingyu Liu, Xinglan Cui, Ying Lv, Chuiyun Tang, Mingjiang Zhang, Hongxia Li, Lang Qiu, Weimin Sun

**Affiliations:** ^1^GRINM Resources and Environment Tech. Co., Ltd., Beijing, China; ^2^National Engineering Laboratory of Biohydrometallurgy, GRINM Group Co., Ltd., Beijing, China; ^3^GRIMAT Engineering Institute Co., Ltd., Beijing, China; ^4^School of Metallurgical and Ecological Engineering, University of Science and Technology Beijing, Beijing, China; ^5^Guangdong Key Laboratory of Integrated Agro-environmental Pollution Control and Management, Institute of Eco-environmental and Soil Sciences, Guangdong Academy of Sciences, Guangzhou, China

**Keywords:** uranium, *Bacillus* spp., bioprecipitation, phosphate-solubilizing bacteria, uranyl phosphate

## Abstract

The remediation of uranium (U) through phosphate-solubilizing bacteria (PSB) is an emerging technique as well as an interesting phenomenon for transforming mobile U into stable minerals in the environment. While studies are well needed for in-depth understanding of the mechanism of U(VI) immobilization by PSB. In this study, two PSB were isolated from a U-tailing repository site. These bacterial strains (ZJ-1 and ZJ-3) were identified as *Bacillus* spp. by the sequence analysis of 16S ribosomal RNA (rRNA) genes. Incubation of PSB in liquid medium showed that the isolate ZJ-3 could solubilize more than 230 mg L^–1^ P from glycerol-3-phosphate and simultaneously removed over 70% of 50 mg L^–1^ U(VI) within 1 h. During this process, the rapid appearance of yellow precipitates was observed. The microscopic and spectroscopic analysis demonstrated that the precipitates were associated with U-phosphate compound in the form of saleeite-like substances. Besides, scanning electron microscopy coupled with energy-dispersive X-ray (SEM-EDS) and Fourier transform infrared spectroscopy (FTIR) analysis of the precipitates confirmed that the extracellular polymeric substances (EPS) might also play a key role in U sequestration. Furthermore, SEM and FTIR analysis revealed that part of U(VI) was adsorbed on the bacterial surface through cellular phosphate, hydroxy, carboxyl, and amide groups. This study provides new insights into the synergistic strategies enhancing U immobilization rates by *Bacillus* spp. that uses glycerol-3-phosphate as the phosphorus source, the process of which contributes to harmful pollutant biodegradation.

## Introduction

Anthropogenic activities such as uranium (U) mining activities, primarily associated with decades of nuclear fuel production and weapon making (Basu et al., 2014), have left considerable hazardous U waste, causing the release of U into the environments. As a radioactive element, U could be accumulated in the human body through the food chain, and its potential chemical and radioactive toxicity can pose adverse effects on the environment and threaten the public health (Pan et al., 2015). Considering its easy migration from waste repositories and long-term contamination of the environment, the presence of U in radioactive wastes has raised global attention. Therefore, it is important to focus on developing efficient remediation and long-term management strategies regarding increasingly severe U contamination.

Researches over the past decade have focused more on U bioreduction processes, which play an important role in U bioremediation (Llorens et al., 2012). However, bioreduction of U(VI) has been shown to occur optimally above pH 7, and its product is easily reoxidized to mobile U(VI) in the presence of oxygen and more slowly oxidized under nitrate-reducing conditions by nitrite or Fe(III) oxyhydroxides (Beazley et al., 2009). Recently, U-phosphate biomineralization has been considered as a potential alternative and an attractive remediation strategy due to its several advantages. Uranyl phosphate minerals are sparingly soluble in a wide range of pH value and remain stable for long periods of time (Beazley et al., 2011). Additionally, compared to U bioreduction process, the process of U-phosphate biomineralization is faster, as it can remove U(VI) from the solution within a week, while U bioreduction process usually needs over 2 weeks. In addition, biological reduction requires the creation of an anaerobic environment, while biomineralization can take place in an aerobic environment. Thus, biomineralization may be suitable for *in situ* and real-time remediation on large areas of hazardous U contaminated waste.

Biomineralization remediation of U-polluted areas by using microorganisms such as phosphate-solubilizing bacteria (PSB) is an emerging area (Choudhary and Sar, 2011; Sousa et al., 2013; Yung and Jiao, 2014; Liang et al., 2015). PSB release organic acids and/or express phosphatase enzymes to liberate inorganic phosphate (Pi) from inorganic phosphate/organophosphate substrates that precipitate U as uranyl phosphate minerals, such as autunite [Ca(UO_2_)_2_(PO_4_)_2_], chernikovite [H_2_(UO_2_)_2_(PO_4_)_2_], saleeite [Mg(UO_2_)_2_(PO_4_)_2_], and ankoleite [K_2_(UO_2_)_2_(PO_4_)_2_] (Song et al., 2019). The biomineralization of U was first observed on *Citrobacter* sp. (Macaskie et al., 1992). Since then, various bacteria, including *Bacillus* sp. (Tu et al., 2019), *Rahnella* sp. (Beazley et al., 2009), *Rhodanobacter* A2-61 (Sousa et al., 2013), *Pseudomonas aeruginosa* (Choudhary and Sar, 2011), and *Acinetobacter* sp. (Sowmya et al., 2014), have been found to be able to solubilize phosphate substrates and therefore enhance the remediation of U with phosphate amendments. Besides, bacteria have high surface-to-volume ratio, and some PSB surfaces have numerous organic functional groups such as phosphate, carboxyl, hydroxyl, and amide groups (Beveridge and Murray, 1980), which can bind U(VI) onto the cell surface.

PSB are ubiquitous in soil. *Bacillus* spp. have the characteristics of phosphate-solubilizing ability, strong environmental tolerance, and large surface area, which contribute to the precipitation of heavy metals (Aka and Babalola, 2016; Song et al., 2019). Notably, *Bacillus thuringiensis* strains, isolated from U-contaminated soil samples in Xinjiang, China, have shown high potential for U(VI) removal and have been used for remediation (Pan et al., 2015). However, the mechanisms of U(VI) immobilization by these microorganisms are inconsistent in a number of existing studies. A previous study (Tu et al., 2019) showed that U could be biomineralized on *Bacillus* sp. dw-2 as UO_2_HPO_4_⋅4H_2_O or (UO_2_)_3_(PO_4_)_2_⋅4H_2_O, while another research implied that U(VI) was initially adsorbed on the *B. thuringiensis* surface and then stabilized by the formation of needle-like amorphous U compounds (Pan et al., 2015).

To date, a majority of studies investigating phosphate-solubilizing bacteria for U remediation have focused on the formation of stable U-phosphate minerals (Sowmya et al., 2014). However, the resistance of PSB to U and the effects of the presence and activity of PSB on U immobilization through synergistic mechanisms are not well understood, impeding the understanding of interactions between U and PSB. In this paper, two indigenous *Bacillus* spp. were isolated from a U-tailing repository site in Fuzhou, Jiangxi province of China, and the interactions between U and *Bacillus* spp. were investigated. The purposes of this study were (1) to isolate U-tolerant PSB and evaluate their phosphate-solubilizing capacity and the process mechanism, (2) to explore the efficacy of U(VI) removal and immobilization ability of *Bacillus* spp., and (3) to study the interaction mechanism between U and *Bacillus* spp. Overall, an environmentally effective method to mitigate U pollution using indigenous PSB is proposed, and synergistic strategies of uranium immobilization by *Bacillus* spp. are postulated. This research broadens the general understanding of U immobilization and can serve as the foundation for future in-depth research on biomineralization processes.

## Materials and Methods

### Materials and Site Description

Samples used in this research were collected from an open-pit U-tailing repository site, located in Fuzhou (27.25°–27.56°N, 115.49°–116.17°E), Jiangxi Province, the southeast of China. This region was one of the main U mining areas in China due to the presence of China’s largest volcanic-type U ore field. The soil samples were collected from 5 to 15 cm of the upper surface in July 2019. The collected samples were immediately stored at approximately 4°C for transportation to the laboratory and remained sealed until bacteria isolation.

### Isolation of Phosphate-Solubilizing Bacteria

Three groups of soil samples (10 g) were cultivated in 100 ml Luria–Bertani (LB) medium after being passed through a 40-mesh sieve and then shaken at 120 rpm for 24 h. In order to isolate the PSB, a serial dilution assay was carried out, and 100 μl of diluted suspension was plated on Pikovskaya’s (PVK) agar medium (Pikovskaya, 1948). Plates were incubated at 30°C for 4–7 days. Later, the colonies were selected from the plates according to the appearance of clear halos around the colonies and were further transferred to the National Botanical Research Institute’s phosphate (NBRIP) growth medium containing (per L) 10 g glucose, 5 g Ca_3_(PO_4_)_2_, 5 g MgCl_2_⋅6H_2_O, 0.25 g MgSO_4_⋅7H_2_O, 0.2 g KCl, 0.1 g (NH_4_)_2_SO_4_, and 2.0% agar (Nautiyal, 1999). After purification, each strain was maintained in nutrient broth (NB) agar and preserved under refrigerated (4°C) condition for further study.

### Molecular Identification of the Isolates

The bacterial strains were identified by 16S ribosomal RNA (rRNA) gene sequencing, after DNA amplification using the universal primers 27F (5′-AGAGTTTGATCCTGGCTCAG-3′) and 1492R (5′-GGTTACCTTGTTA CGACTT-3′) as previously described by Das et al. (2014). Prior to sequencing, PCR products were purified with Gel Extraction Kit (Sangon, China) as per the manufacturer’s instructions. Purified PCR products of 16S rRNA gene were sequenced, analyzed, and submitted to the National Center for Biotechnology Information (NCBI) GenBank database using BLASTN algorithm to identify the most similar 16S rRNA gene. Multiple sequence alignment and data analysis were conducted using MEGA 6.0 software, and a phylogenetic tree was constructed by neighbor-joining method. Nucleotide sequences of the two isolates ZJ-1 and ZJ-3 used in this study were then deposited in GenBank (NCBI) with accession numbers MW793405 and MW793406, respectively.

### Evaluation of Phosphate-Solubilizing Ability of PSB Strains

The PSB isolates were further studied using insoluble Ca_3_(PO_4_)_2_ in NBRIP broth medium for their ability to solubilize P. Two milliliters preinoculum of PSB grown for 24 h in liquid nutrient medium was transferred to a 250-m Erlenmeyer flask containing 100 ml NBRIP broth medium and then incubated for 14 days at 30°C. Sterile NBRIP medium served as the control in these experiments. The initial pH of the medium was adjusted to 6.5. All experiments were performed in triplicate, and the errors were calculated to be < 5%. The homogenized suspensions were collected periodically to assess pH and P concentrations (Teng et al., 2019a). The PSB cultures were filtered [0.22 μm, polyvinylidene fluoride (PVDF)] prior to P concentration analyses. The concentration of P released into the solution was measured by the molybdenum-blue method of Murphy and Riley (1962).

### Bioprecipitation of U

Experiments with two phosphate-solubilizing bacterial strains were conducted at pH 7.0 (adjusted with 0.1 M NaOH or 0.1 M HCl). Triplicate flasks each containing 100 ml NBRIP liquid medium supplemented with 10 mM glycerol-3-phosphate (G3P) as the phosphorus source were inoculated with approximately 10^8^ cells ml^–1^. Prior to inoculation, two strains were grown in NB broth at 30°C for 24 h. Cells in logarithmic phase were harvested by centrifugation (5,000 rpm for 20 min), then washed twice with isotonic saline solution (0.85% NaCl), and gently resuspended in liquid medium. A control experiment was also performed in parallel with uninoculated medium. All flasks were incubated at 30°C with shaking at 150 rpm, then amended with U (50 mg U L^–1^ as uranyl nitrate solution) and 10 mM NaHCO_3_ after 35 h of incubation to minimize loss of cell viability due to U toxicity. Subsamples were aseptically withdrawn at regular intervals to determine (i) optical density at 600 nm (OD_600_), (ii) orthophosphate concentration, (iii) pH, and (iv) soluble U concentrations. Besides, bacterial cells in the supernatant were harvested by centrifugation to evaluate the effect of U on cell morphologies and functional groups, and the yellow precipitates formed during the reaction were collected to analyze the solid elemental composition through a scanning electron microscope equipped with energy dispersive X-ray spectroscopy (SEM-EDS).

### Analytical Techniques

#### The Quantification of Uranyl

The concentration of dissolved U(VI) was analyzed by a colorimetric technique by Tu et al. (2019), using a UV-Vis spectrophotometer TU-1810 (Beijing Persee, China). In brief, 1 ml sample was mixed with 1 ml of 0.05% Arsenazo(III) and 8 ml chloroacetic acid–sodium acetate (CH_2_ClCOOH–CH_3_COONa) buffer. After 30 min standing, the absorbance at 652 nm was measured.

#### Fourier Transform Infrared Spectrometer

Fourier transform infrared (FTIR) spectrum was obtained to elucidate the specific functional groups involved in U immobilization. The bacterial cells in the supernatant and yellow precipitates in the solution were centrifuged at 8,000 rpm (15 min, 4°C). The samples were washed twice with 0.1 M NaCl solution, then lyophilized and grinded. The dried samples were prepared in a KBr pellet with a sample/KBr ratio of 1:100, and thin pellets were analyzed by an FTIR spectrometer (Thermo Fisher, Nicolet iS5, Waltham, MA, United States), using an IR beam, scanning wavenumber range from 4,000 to 450 cm^–1^, and a scanning accuracy of 4 cm^–1^.

#### Scanning Electron Microscope Equipped With Energy Dispersive X-Ray Spectroscopy

SEM-EDS techniques were used to analyze bacterial cells before and after exposure to U. Bacterial cells were recovered by centrifugation (5,000 rpm, 20 min), washed twice in 5 mM piperazine-N,N′-bis(2-ethanesulfonic acid) (PIPES) buffer (pH 6.5). The bacterial samples were fixed overnight (at 4°C) in 2.5% glutaraldehyde and then washed three times with the same buffer. Then, cell pellets were dehydrated with a graded series of ethanol (30, 50, 70, 85, and 100%) for 15–20 min. Finally, the pellets were lyophilized in a freeze dryer, coated with gold–palladium and analyzed by using a scanning electron microscope (SEM) (SU8010, Hitachi, Japan) (Sharma et al., 2018). EDS analyses were performed at 5 kV with the SEM fitted with Oxford EDS detector and Gatan CCD camera (X-MaxN, Oxford, United Kingdom).

#### Statistical Analysis

All the experiments were carried out in triplicate. Means for different treatments were compared using a one-way analysis of variance (ANOVA). The selected significance level was 95% (i.e., *p*-value of 0.05).

## Results

### Isolation and Taxonomic Identification of Phosphate-Solubilizing Bacteria

The phosphate-solubilizing bacteria were isolated from radioactive uraniferous waste. More than 30 morphologically distinct strains were obtained on PVK medium. Only two bacteria (named as ZJ-1 and ZJ-3) that showed obvious colonies with clear halos were selected to purify on NBPIR agar medium as potential phosphate-solubilizing bacteria. Based on the classical Gram’s staining methods, the two PSB isolates selected above were shown to be Gram positive. The cells of ZJ-1 were short rod-shaped, yellowish, with a smooth and shiny surface, and ZJ-3 cells were long rod-shaped, yellowish, rough, and opaque.

**FIGURE 1:**
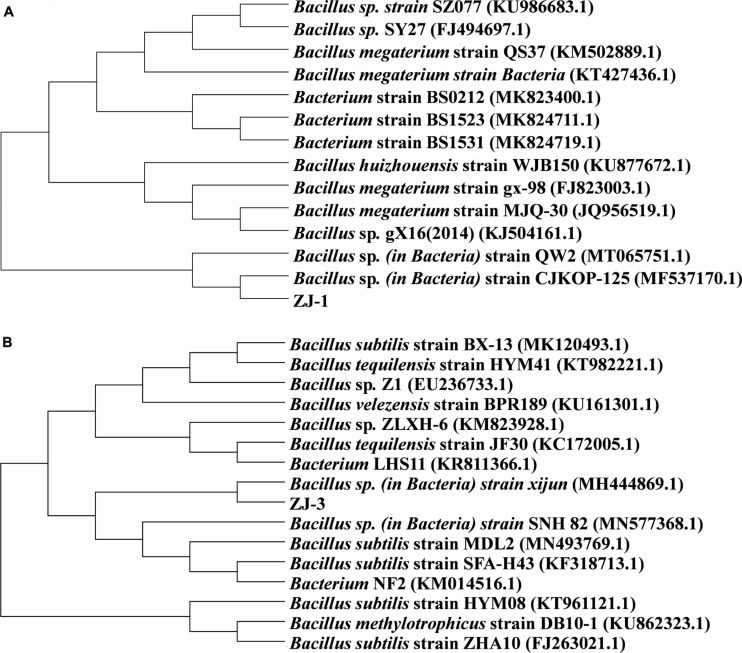
Phylogenetic tree based on 16S rRNA gene sequences of isolated **(A)** ZJ-1, **(B)** ZJ-3, and closely related bacteria.

The BLAST searches revealed that the 16S rRNA gene of ZJ-1 both shared 100% similarity to those of *Bacillus megaterium* strain MJQ-30 and *Bacillus huizhouensis* strain WJB150. To further explore the taxonomic position and relationships, the phylogenetic tree based on the 16S rRNA gene of ZJ-1, MJQ-30, and WJB150 and the phylogenetically related strains from NCBI database by neighbor joining method was constructed; the result is shown in Figure 1A. The phylogenetic tree revealed that the isolate ZJ-1 belonged to the genus *Bacillus*. Based on a sequence identity of 97% or more similarity to *Bacillus* sp. *strain xijun* and *Bacillus subtilis* strain MDL2, ZJ-3 isolates were closely related to the genus *Bacillus* (Figure 1B).

### Evaluation of Phosphate-Solubilizing Characteristics of the Two Isolates

**FIGURE 2:**
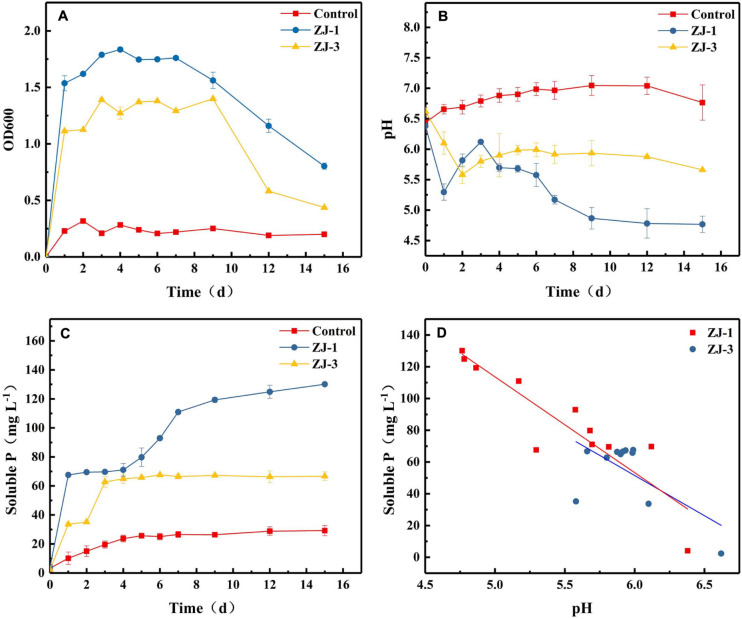
The phosphate-solubilizing properties of PSB strains in NBRIP medium, demonstrated by orthophosphate release from Ca_3_(PO_4_)_2_; **(A)** OD600, **(B)** pH, **(C)** the solubilized P content, and **(D)** the relationship between pH and solubilized P content.

Figure 2 summarizes P solubilization during incubations of ZJ-1 and ZJ-3 in NBRIP liquid medium containing Ca_3_(PO_4_)_2_ as the sole P source. The OD600 values of both cultures exceeded 1.0 after 1 day of incubation, indicating a fast bacterial growth in the NBRIP liquid medium (Figure 2A). After 7–9 days, the OD600 in both PSB cultures started to decline. Figure 2B shows the pH of PSB isolates during 15 days incubation. The pH values initially significantly dropped, decreasing from 6.50 to 5.30 and 5.58 in ZJ-3 and ZJ-1 cultures, respectively. The pH increased steadily between days 1 and 3 of the ZJ-1 incubation, after which it started to drop again. After the initial decrease, the pH of ZJ-3 increased slightly from day 2 and plateaued after a few more days of incubation. It was observed that the solubilization of tri-calcium phosphate (Figure 2C) was accompanied by a significant decline in pH of the culture (Figure 2B). Both bacterial strains exhibited higher phosphate-solubilizing capacity compared to the uninoculated control. The concentration of dissolved phosphate was significantly increased after 1 day in ZJ-1 culture and continued to rise (although slowly). Maximum phosphate solubilization (130.13 mg L^–1^) was observed on day 15 when the maximum pH drops (4.77) occurred. The ZJ-3 culture showed a lower ability of solubilizing P from insoluble Ca_3_(PO_4_)_2_ compared to ZJ-1. The maximum dissolved P concentration reached 62.73 mg L^–1^ after 3 days of incubation, after which it stayed constant, correlating well with only marginal changes in pH.

The results indicated that phosphate solubilization by PSB increases with decreasing pH of the culture (Figures 2B,C). Therefore, the correlation between pH and solubilized P concentration was investigated (Figure 2D). Pearson correlation analysis showed that there was a negative linear correlation between the amount of dissolved phosphate and pH in the culture of strain ZJ-1 (*R*^2^ = 0.82, *P* < 0.05), and no significant correlation was found in the culture of strain ZJ-3 (*R*^2^ = 0.41, *P* > 0.05). This result implies that the mechanisms of P solubilization may be different between ZJ-1 and ZJ-3.

### U Bioprecipitation by the Isolates

**FIGURE 3:**
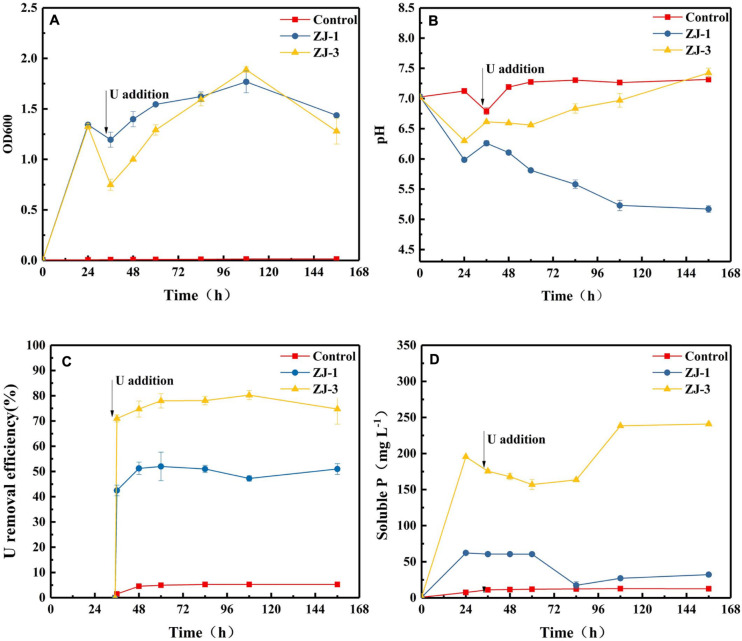
PSB strains were incubated to evaluate G3P metabolism with concomitant U(VI) precipitation; **(A)** optical density at 600 nm (OD_600_), **(B)** pH, **(C)** U removal efficiency, and **(D)** phosphate concentrations.

Incubations with strains of ZJ-1 and ZJ-3 were conducted at pH 7 in the presence of 10 mM G3P with 50 mg L^–1^ UO_2_(NO_3_)_2_ added at 35 h. Figure 3A shows the effects of U on the growth of the two isolates. In the absence of U, cell densities of the two isolates were increased quickly at the first 24 h of incubation. Within 1 h of U addition, the OD dropped from ∼1.34 to 1.20 and 0.75 in ZJ-1 and ZJ-3 cultures, respectively. The U(VI) had a lower inhibitory effect on the growth of ZJ-1 than that of ZJ-3. However, the OD600 of both strains increased again once U was gradually removed from solution. At the end of the incubation, OD600 in the cultures were comparable to their initial values. Besides, the OD600 values began to drop when the reaction time exceeded 108 h, which means the cells were dying off in the stationary growth phase, and the active growth phase should be a response to the U presence.

The addition of U(VI) to phosphate-containing cultures with the isolates resulted in the immediate formation of yellow precipitate with corresponding decrease in concentrations of U and phosphate. The changes in pH, U(VI) removal efficiency, and soluble P concentration in the cultures as a function of contact time are shown in Figure 3. Different pH trends were observed in different cultivation groups. The pH of the cell-free control was almost unchanged. Noticeably, the pH of the assays with strain ZJ-1 gradually decreased from 6.26 to 5.17, while the pH of the assays with strain ZJ-3 increased from 6.62 to 7.43 after U addition. This indicated that the phosphate solubilization in ZJ-3 was not caused by acid production as in ZJ-1 but by enzymatic activity (i.e., alkaline phosphatase). Significantly higher concentration of orthophosphate was released by ZJ-3 than ZJ-1 within 156 h of incubation. Subsequently, both groups showed a reduction in orthophosphate concentration due to the addition of 50 mg L^–1^ U. The U removal efficiency was approximately 5% in the control sample, and it kept stable throughout the time course of the experiment. In ZJ-3 incubations, 71% U was removed from the solution within 1 h, accompanied by the decrease in orthophosphate concentration from 195.5 to 175.5 mg L^–1^. After 108 h, up to 80% of the U was removed, and the inorganic P concentration gradually increased to 238.5 mg L^–1^. The removal efficiency of U by ZJ-1 was significantly lower than that of ZJ-3. At the initial stage only 42.5% reduction with the addition of U was observed, and the maximum removal rate by strain ZJ-1 was approximately 52%.

### The Analysis of U-Loaded Microbes

#### FTIR Analysis

**FIGURE 4:**
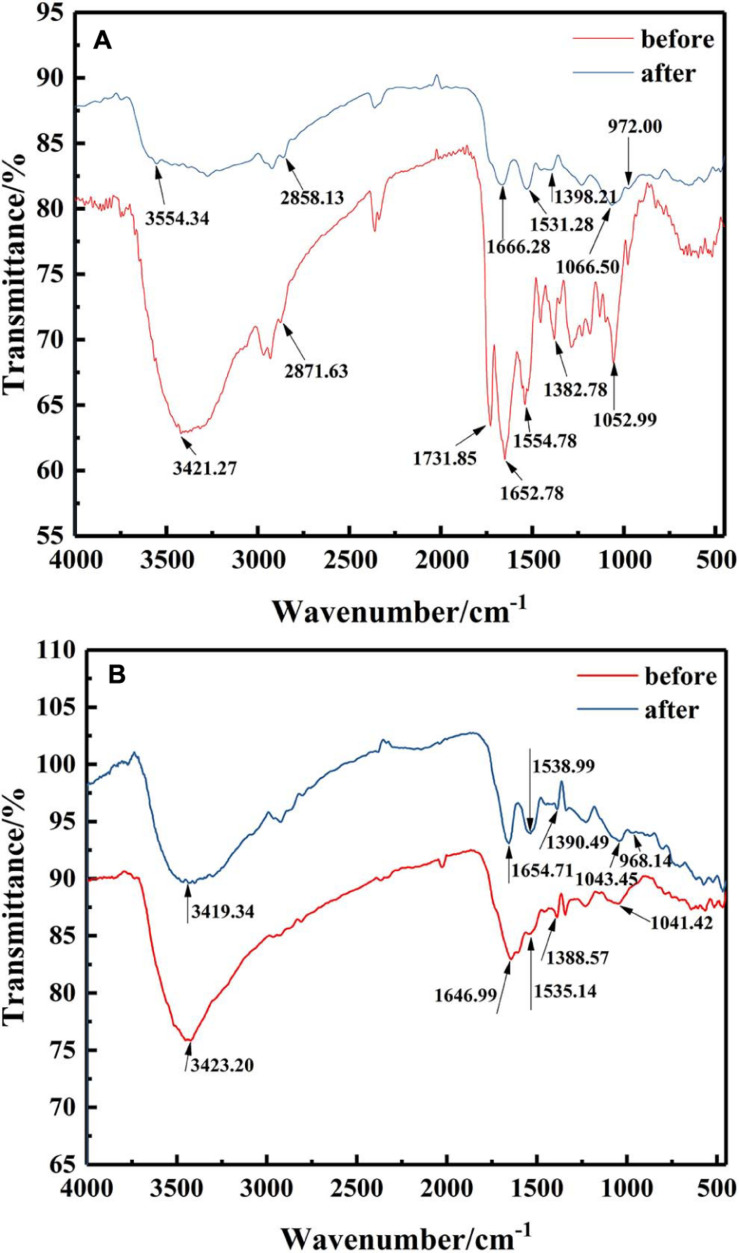
FTIR spectra of the mineralized products by **(A)** ZJ-1 and **(B)** ZJ-3 before and after U treatment (initial concentration, 50 mg L^–1^ of U at pH 7.0; time 7 days; temperature, 30°C).

To elucidate the functional groups involved in U binding on the microbial surface, an FTIR spectrum was obtained for the isolates with or without U treatment from 4,000 to 450 cm^–1^ (Figure 4). FTIR spectra revealed that the main functional groups were observed on the cell surface, like hydroxyl groups (the peak at 3,200–3,600 cm^–1^), amino group (the peak around 1,660 and 1,535 cm^–1^), phosphate group (the peak around 1,062 cm^–1^), and carboxyl group stretching vibration at 1,300–1,420 cm^–1^. These might be related to the immobilization of U by *Bacillus* sp. (Pagnanelli et al., 2000; Doshi et al., 2007; Choudhary and Sar, 2011).

After exposure to U, it could be seen from the FTIR spectra that most of those peaks showed significant changes when compared with U-free control group. The main performance was that all the peak intensities were weakened, and positions were shifted. As depicted in Figure 4A, after interaction with U, the observed shifts from 3,421.27 cm^–1^ to high wavenumber of 3,554.34 cm^–1^, even the broader peak shape, both indicated that –OH and –NH_2_ were involved in binding of U. No major changes were observed at 2,871.63 cm^–1^. The peak around 1,731.85 cm^–1^ disappeared after interaction, suggesting that most of the aldehyde groups interacted with U (Wang et al., 2019). Significant shifts of 1,652.28 and 1,554.78 cm^–1^ suggested that amide group in the protein may be a main contributor to the bioremoval of U. The observed shifts around 1,382.78 cm^–1^ also indicated that carboxyl groups were involved in U binding. Peak intensity shifted from 1,052.99 to 1,066.50 cm^–1^, suggesting the interaction between U and functional groups on cell surface, such as proteins and phosphate groups. It is worth noting that the appearance of an obvious new peak at 972 cm^–1^ was assigned to the stretching of U = O on the surface (Kazy et al., 2009). Besides, according to Figure 4B, comparing the isolates of ZJ-3 before and after exposure to U, main changes in the FTIR spectra occurred at the wavenumbers of 3,423.20, 1,646.99, and 968.14 cm^–1^. Overall, the differences between the two curves (before and after) were less than those of ZJ-1. In addition, the peaks 1,535.14, 1,388.57, and 1,041.42 cm^–1^ drifted to 1,538.99, 1,390.49, and 1,043.45 cm^–1^, respectively, which may be caused by the changes in other groups nearby.

#### SEM-EDS Analysis

**FIGURE 5:**
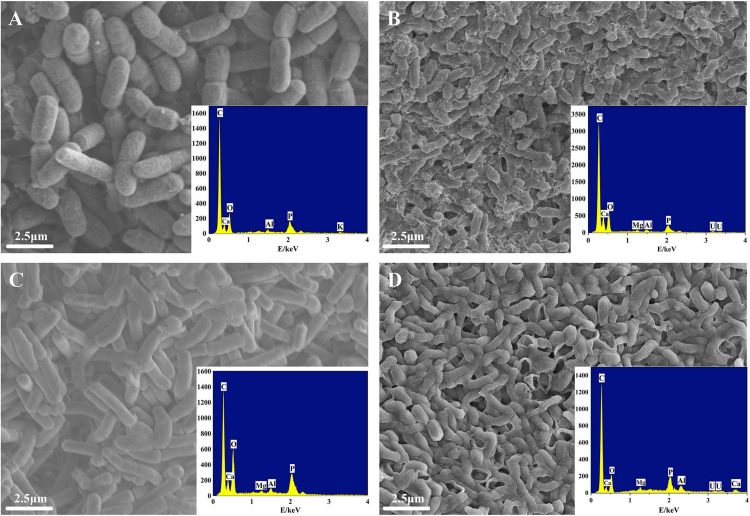
SEM-EDS images of two isolates before and after exposure to U(VI): **(A)** ZJ-1 before exposure to U(VI), **(B)** ZJ-1 after exposure to U(VI), **(C)** ZJ-3 before exposure to U(VI), and **(D)** ZJ-3 after exposure to U(VI).

Cell morphology changes of ZJ-1 and ZJ-3 treated with or without U(VI) were revealed by a set of SEM images with EDS analysis (Figures 5A–D). Initially, the two isolates had exhibited that the bacteria had smooth surface and intact shape (Figures 5A,C). After interacting with U(VI), the bacterial surfaces of ZJ-1 and ZJ-3 were no longer smooth, and the cells became rough and shrunk to some extent (Figures 5B,D). In addition, a large number of mineral-like deposits were observed on the surface of microbial cells. Combining with the FTIR results, the formation of these sediments may be due to the functional groups on the surface of bacteria, such as phosphoric acid group and amide group (Song et al., 2019). Meanwhile, in order to identify the deposited elements on microorganism cells, EDS analyses were conducted simultaneously. Results showed that specific peaks corresponding to carbon (C), phosphorus (P), oxygen (O), and U appeared after exposure to uranyl ions. Cell debris is known to be rich in organic matter containing O, C, and P, so the U observed simultaneously proved to be adsorbed by the functional groups on the cell surface.

### The Analysis of Microbially Mediated U Precipitates

**FIGURE 6:**
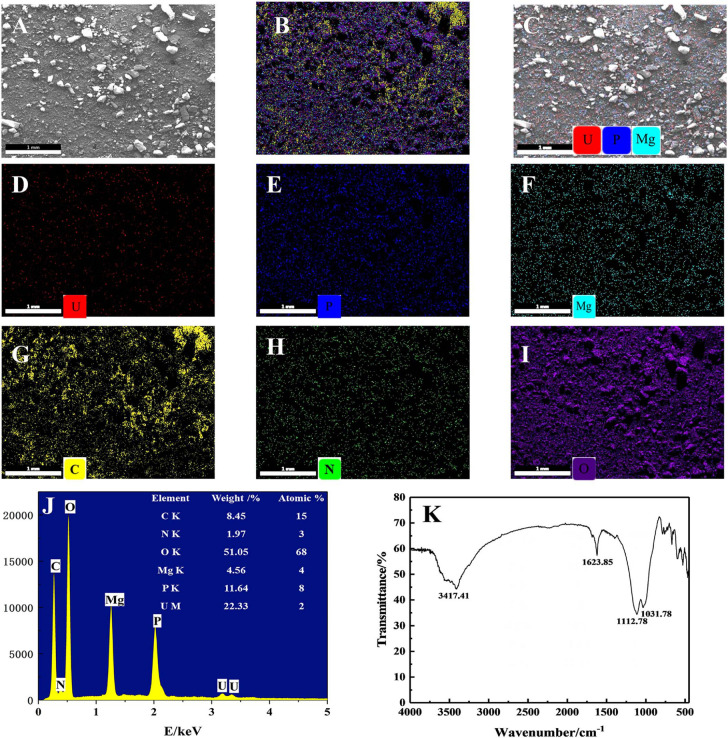
SEM-EDS and FTIR spectra analyses of the precipitates; **(A)** SEM images of U phosphates precipitates and **(B)** their corresponding EDS maps with the distribution of **(C)** P + U + Mg + C + N + O, **(D)** P + U + Mg, U, **(E)** P, **(F)** Mg, **(G)** C, **(H)** O, and **(I)** N. **(J)** EDS results of the precipitates and **(K)** FTIR results of the precipitates.

The yellow precipitates retrieved from solutions were analyzed by SEM-EDS to ascertain the elements involved in the U precipitation (Figure 6). Figures 6A,B show the SEM images and EDS analysis of the precipitation. After interaction with U, it could be clearly seen from Figure 6C that a dense red U signal and blue P signal uniformly distributed on the surface, indicating that precipitates were composed of U and P. As shown in Figure 6J, the precipitates were detected to contain elements such as C, O, N, P, Mg, and U. Other substances such as EPS may also be contained in the precipitates when collecting the precipitated products of U(VI), as EDS spectra of the precipitates revealed the existence elements of O, N, and C, which is consistent with the composition of EPS (Wang et al., 2019). Besides, the presence of U, Mg, and P suggests that the yellow precipitate may be U–P associated with Mg, saleeite-like in character. In addition, as shown in Figure 6K, FTIR spectra of the precipitates revealed signals at 3,417.41 cm^–1^, which may be attributable to the hydroxyl groups. The noticeable absorbance band at around 1,623.85 cm^–1^ was assigned to be amide groups. The band between 1,200 and 900 cm^–1^ resulted from the C-O-P stretching of polysaccharides.

## Discussion

### Screening and Identification of the Isolates

The presence and distribution of microbes are often site specific; thus, U tailings were chosen to isolate PSB due to greater possibility of occurrence of U-resistant bacteria. In this study, two phosphate-solubilizing bacterial isolates were screened from U tailings based on the above idea. Referring to 16S rRNA gene sequence analysis, the isolated strains ZJ-1 and ZJ-3 belonged to the genus *Bacillus*. The bacterial isolates, ZJ-1 and ZJ-3, exhibited maximum phosphate solubilization of 66.8–130 mg L^–1^, respectively. In a previous report, Audipudi et al. (2012) found that seven bacterial isolates, including *Bacillus subtilis*, *Pseudomonas* sp., and *Azotobacter* sp., isolated from the soil of Chollangi mangrove environment, exhibited phosphate-solubilizing ability in the range of 80–100 mg L^–1^ when using calcium phosphate as the single P source. In an earlier report, much higher phosphate-solubilizing activity (523.69 mg L^–1^) was also observed by the PSB isolated from calcareous rhizosphere soils (Liu et al., 2015).

Presenting a promising bioremediation strategy, PSB can release phosphate from insoluble P sources by various mechanisms. In this study, it was observed that the concentration of released phosphate corresponded to the decrease in pH during the bacterial growth. This was supported by a linear relationship between the concentrations of solubilized P and pH of ZJ-1 strain (*R*^2^ = 0.82). The results were similar to the findings of Park et al. (2011), who reported that the simple regression analysis between solubilized P and pH (*R*^2^ = 0.79) showed a significant linear relationship. The results suggested that the processes resulting in phosphate solubilization may involve several different reactions (Bashan et al., 2013):

(1) The simple acidification of the medium because of proton release (i.e., NH_4_^+^ assimilation), resulting in the dissolution of phosphate. The reaction equation, Equation (1), is as follows:

(1) $${\text{Ca}}_{3}{\left({\text{PO}}_{4}\right)}_{2}+2{\text{H}}^{+}\to 2C{\text{aHPO}}_{4}+{\text{Ca}}^{2+}$$

(2) The pH decrease resulting from the release of organic acid could be related to P solubilization, as demonstrated in the reaction equation, Equation (2).

(2) $$\begin{array}{c} {\text{Ca}}_{3}{\left({\text{PO}}_{4}\right)}_{2}+3m{\text{H}}_{\text{n}}\text{X}\to \text{m}{\left[{\text{CaX}}_{\text{m}}\right]}^{2-\mathrm{mn}}+2{\text{HPO}}_{4}^{2-}+\\ \left(3mn-2\right){\text{H}}^{+} \end{array}$$

where H_*n*_X is a complexing acid, m is the stoichiometric coefficient in the calcium complex formed, and n is the index equal to the absolute value of the charge of the complexing anion (X^*n*–^).

There was no significant correlation between pH and P in the assays with strain ZJ-3 (*R*^2^ = 0.41), which suggested that the P solubilization in the solution was not merely caused by the decrease in pH value, but other mechanisms also occurred in the system. There must be some other mechanisms occurring in this system. Researchers concluded that there are three major mechanisms of P solubilization. First is the release of organic acids. PSB can synthesize organic acids to reduce pH, which in turn increases P solubilization. Behera et al. (2017) indicates that various organic acids, such as malic acid, lactic acid, and acetic acid were detected in the *Serratia* sp. broth culture. Second is the extracellular enzymes release. Microorganisms can produce various enzymes, such as phytase, C–P lyase, nuclease, and phosphatase to accelerate the dissolution of inorganic phosphate release (Achal et al., 2007). Third is the release of protons originating from respiration or NH_4_^+^ assimilation (Illmer and Schinner, 1995).

### U Removal Efficiency and Immobilization Ability by Isolates

No biological function is known for U, and it is considerably more toxic compared to other heavy metals. In presence of U, bacterial growth and metabolic processes may be severely impeded. The active ZJ-1 and ZJ-3 bacterial cells were incubated in the presence of U, significant resistance to U was observed through survivability (OD_600_) and cell morphology (SEM) analyses. During the first several hours of U addition to the medium, cell growth and lysis were restrained, but the growth recovered over time. The SEM results also showed that most cell walls were intact after they were in contact with 50 mg L^–1^ U. The isolates exhibited U tolerance, probably due to their adaptation to U as these strains were isolated from U tailings soil. A previous study also identified that a *Pseudomonas aeruginosa* strain isolated from U mine waste showed U resistance and accumulation following incubation in 100 mg L^–1^ U for 6 h at pH 4.0 (Choudhary and Sar, 2011). The bacterial strains were isolated from a U-contaminated soil and could survive in the presence of 50 mg L^–1^ U, which was much higher than that in most other U-contaminated environments (<5 mg L^–1^ U). These U-resistant isolates could therefore be used to stabilize U in highly contaminated environments. In general, the application of indigenous bacteria in contaminated sites can greatly improve the efficiency of remediation.

A large number of microorganisms have been reported to efficiently precipitate U when supplied with glycerol phosphate, such as *Serratia* species (Newsome et al., 2015a), *Caulobacter crescentus* (Yung and Jiao, 2014), *Bacillus*, and *Rahnella* species (Martinez et al., 2007). To test whether the two isolates ZJ-1 and ZJ-3 were responsible for U biomineralization, cells were resuspended in a certain volume of U(VI) and glycerol-3-phosphate solution. The results clearly demonstrated the U biomineralization activity of strains ZJ-1 and ZJ-3, as evidenced by the decrease in U concentration and increase in insoluble U over time. Notably, although the ZJ-3 strain exhibited much higher pH value than ZJ-1, ZJ-3 is still solubilizing more phosphate than ZJ-1. As a result, the efficiency of U precipitation in ZJ-3 was higher than ZJ-1. It has been shown that glycerol phosphate biodegradation involves a bacterial phosphatase in *Serratia* sp., which catalyzes the hydrolytic cleavage of the C-P bond and rapidly releases inorganic phosphate, which then precipitates with the U(VI) (Newsome et al., 2015b). Whether ZJ-3 possesses such enzymatic activity needs further investigation. A comparison of U removal efficiency with and without bacteria suggested that microbial activities were essential for biomineralization. Beazley et al. (2009) also demonstrated that U biomineralization was promoted by microbial phosphatase activity. Altogether, these incubations suggest that the isolated *Bacillus* spp. can survive well in the presence of U and soluble sufficient organophosphate to precipitate U(VI). These findings demonstrate the potential applicability of the isolates PSB in U contaminated environments.

### Possible Immobilization Mechanisms of *Bacillus* spp. Interaction With U

In this study, PSB isolated from U tailings exhibited intrinsic abilities to remove U from solution. Based on all above-mentioned analyses, we proposed the possible U(VI) immobilization mechanism by *Bacillus* spp.

*Bacillus* spp. could adsorb U(VI) in a short time, leading to a rapid capture of U(VI) from the water. The SEM-EDS observations proved that U could be absorbed on the cell surface, as many U-bearing tiny particles were observed on the bacterial surface (Figures 5B,D). Nie et al. (2017) have also reported the similar finding that the tiny nanolamellar crystal was detected on the *D. radiodurans* surface when reacted with 100 mg L^–1^ U at pH 5 after 1 day. The biosorption of U on the cell surface is known as an extremely rapid process (Wang et al., 2017). The basis of the adsorption mechanism is the rich and various functional groups on bacterial cell surface, which provide abundant sites to bind cations/anions (Bader et al., 2018). Thus, FTIR analyses were used in this paper to further verify that bacterial cell wall was one of the major sorption sites, and hydroxyl, amide, aldehyde, carboxyl, and phosphate groups belonged among the major binding groups. Meanwhile, we also found a distinct peak at 972 cm^–1^ as evidence of the presence of uranyl group on the cell surface. This finding was in agreement with the study of Sowmya et al. (2014). Several works also described the ability of *Bacillus* sp. to adsorb U on the cell surface. For example, Zhao et al. (2016) reported the biosorption and bioaccumulation ability of *Bacillus* sp.dwc-2 to U.

Moreover, the bioprecipitation process of U(VI) by *Bacillus* spp. also played an important role in U removal. The immediate decrease in soluble P concentration and the occurrence of yellow precipitates on addition of U suggests that phosphate could sequester U as U–P precipitates. The products of U precipitation varied in different studies (Song et al., 2019). Researchers proposed that microorganisms could transform the precipitated U into crystalline chernikovite, while others demonstrated the potential for U(VI) removal from solution via precipitation of U(VI) as autunite, sodium–autunite, meta-ankoleite, and uramphite (Zheng et al., 2017). In this study, we found that U could be precipitated in the presence of glycerol phosphate with PSB, leading to the formation of U phosphate biominerals. The EDS spectra suggested that the yellow precipitates may be formed as saleeite, which does not tally with earlier reports. This phenomenon probably occurred due to the use of medium rich in Mg^2+^ rather than Ca^2+^.

EPS are metabolic products that accumulate on the surface of microbial cells and provide protection by reducing chemical exposure and stabilizing the membrane (Teng et al., 2019b). Previous studies have shown that EPS rich in functional groups and proteins were highly reactive with uranyl ions, suggesting that EPS could be used for rapid U adsorption (Cao et al., 2011; Li et al., 2018). In this paper, our results indicated that the EPS generated by *Bacillus* spp. may also participate in the sequestration of U. The FTIR analysis proved that the precipitates contained active functional groups (-COOH, -OH, C-O-P, -CO-N, and so on), which was consistent with the feature of EPS (Teng et al., 2019b; Wang et al., 2019). In addition, EPS are primarily composed of proteins, carbohydrates, nucleic acids, and heteropolymers, which are usually consisted of elements such as C, N, and O (Wei et al., 2017). The SEM-EDS analysis further confirmed the presence of O, C, and N in the precipitates (Figure 6J).

**FIGURE 7:**
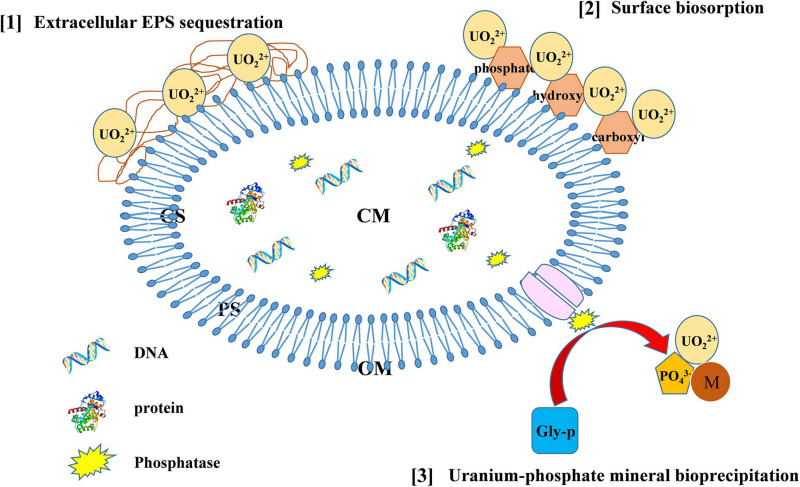
The possible mechanisms of U immobilization by *Bacillus* spp. (CS, cytoplasm; CM, cytomembrane; PS, periplasm; OM, outer membrane).

Based on the above-mentioned analyses, we proposed strategies of *Bacillus* spp. immobilization U (as delineated in Figure 7). U-resistant *Bacillus* spp. possess various strategies to immobilize U, including U–phosphate mineral bioprecipitation, surface biosorption, and EPS sequestration, which may serve as potential methods for bioremediation of U-contaminated sites and be worthy of further in-depth studies.

## Conclusion

In this study, we have investigated the immobilization mechanisms between U and *Bacillus* spp., isolated from U tailing repository site in Southeast China. The isolates could survive well and dissolve phosphate at significant concentrations of 50 mg L^–1^ U. Maximum phosphate was solubilized by strain ZJ-3 with a production of 238.5 mg L^–1^ soluble phosphate. Results revealed that *Bacillus* spp. showed good potential for the application in removal of U from aqueous solutions, and approximately 81.25% of U(VI) was removed rapidly in a relatively short remediation time, represented in a form of yellow precipitate. The results of microscopic and spectroscopic analyses demonstrated that the precipitates were a type of saleeite-like U–phosphate compound, and *Bacillus* spp. could immobilize U through enzymatic bioprecipitation, cell surface biosorption, and EPS sequestration. Overall, this work provides new insights into the interaction mechanisms between U and PSB, suggesting that PSB, particularly U-resistant strains isolated from contaminated sites, could be used for bioremediation of U-contaminated environments.

## Data Availability Statement

The datasets presented in this study can be found in online repositories. The names of the repository/repositories and accession number(s) can be found below: GenBank with accessions SUB9339268 78_(ZJ-1)_31219070100550 MW793405 and SUB9339268 78_(ZJ-3)_31219070100550 MW793406 (https://submit.ncbi.nlm.nih.gov/subs/?search=SUB9339268).

## Author Contributions

XL provided the idea of this work. JZ performed the experiments, collected samples, detected and analyzed the data, prepared the figures, and wrote the manuscript. XH detected physiochemical properties. MZ and XC were involved in experimental design. CT and HL collected samples. YL, LQ, and WS revised the manuscript. All authors contributed to the article and approved the submitted version.

## Conflict of Interest

JZ, XH, XL, XC, YL, CT, MZ, and HL were employed by company GRINM Group Co., Ltd. The remaining authors declare that the research was conducted in the absence of any commercial or financial relationships that could be construed as a potential conflict of interest.
